# Measurement of intrapleural pressure in patients with spontaneous pneumothorax: a pilot study

**DOI:** 10.1186/s12890-019-1038-9

**Published:** 2019-12-30

**Authors:** Hiroyuki Kaneda, Takahito Nakano, Tomohiro Murakawa

**Affiliations:** 1 Division of Thoracic Surgery, Kansai Medical University Medical Center, 10-15 Fumizonocho, Moriguchishi, Osaka, 570-8507 Japan; 2 Department of Thoracic Surgery, Kansai Medical University, Hirakatashi, Osaka, Japan

**Keywords:** Spontaneous pneumothorax, Initial management, Intrapleural pressure, Needle aspiration, Chest tube drainage

## Abstract

**Background:**

The initial management of pneumothorax remains controversial, and we speculated that this might be because there is no method available for evaluation of air leak during initial management. We have developed a system for measurement of intrapleural pressure in pneumothorax to address air leak without the need for chest drainage. The aim of this clinical study was to confirm the ability of this measurement system and to determine the clinical impact of management of air leak.

**Methods:**

Patients in whom need aspiration was indicated for spontaneous pneumothorax were enrolled in the study. The intrapleural pressure was measured during stable breathing and data recorded when patients were coughing were excluded.

**Results:**

Eleven patients were enrolled in the study between December 2016 to July 2017. The patterns in change of intrapleural pressure varied widely depending on the state of the pneumothorax. The mean intrapleural pressure values on end-inspiration and end-expiration in patients with persistent air leak was significantly lower than those in patients without persistent air leak (*p* = 0.020). The number of negative mean pressure recordings in end-inspiration and end-expiration was significantly lower in patients with persistent air leak than in those without persistent air leak (*p* = 0.0060).

**Conclusions:**

In this study, we demonstrated that intrapleural pressure could be successfully measured and visualized in patients with pneumothorax. Whether or not the pressure value is a predictor of persistent air leak needs to be confirmed in the future.

## Background

The treatment of spontaneous pneumothorax has been debated for at least 80 years [1, 2]. Pneumothorax is defined as the presence of air in the pleural cavity. Objective evaluation of pneumothorax is mainly performed by chest radiography and computed tomography (CT) [3]. Unfortunately, these imaging techniques only provide static images and cannot confirm an air leak, which is the cause of spontaneous pneumothorax. When patients undergo interventional drainage with a chest tube, movement of water in the chamber of a drainage bag or, more recently, a digital system, can show intrapleural pressure. However, it is not easy to determine whether a patient has an air leak unless drainage is performed. Several approaches can be used to address this question in the absence of drainage, i.e., suspicion based on clinical examination findings, such as symptoms and oxygenation, a change in lung collapse seen on chest radiography over time, and measurement of intrapleural pressure [4].

The initial management of pneumothorax remains controversial [5], and we speculated that this might be because there is no method that is clinically available for evaluation of air leak during initial management. We have developed a system for measurement of intrapleural pressure in patients with pneumothorax to address air leak without the need for chest drainage. This system measures intrapleural pressure using a portable visual device. We have verified the system in a handcrafted model of the thoracic cavity using a polyethylene terephthalate bottle and two balloons (Additional file 1: Figure S1a). We confirmed that the change in air pressure ranged from 0 cmH_2_O to − 20 cmH_2_O and showed a continuous periodic curve (Additional file 1: Figure S1B). A model of progressive pneumothorax was created by making a small hole in the lung balloon to simulate a lung injury. The pressure in the simulated thoracic cavity was observed to increase gradually in accordance with manual movement of the diaphragm balloon, which was used as a model of tension pneumothorax (Additional file 1: Figure S1C). Next, we verified the system by measurement of intrapleural pressure in a pig model (Additional file 2: Figure S2).

The aim of this clinical study was to confirm the ability of this measurement system to identify air leak and to determine the clinical impact of management of air leak in patients with spontaneous pneumothorax.

## Methods

The study was approved by the Institutional Review Board of Kansai Medical University (approval date: December 6, 2016; approval number: 1648) and was performed in accordance with the principles of the Declaration of Helsinki. Written informed consent was obtained from all patients included in the study. Patients in whom aspiration was indicated for spontaneous pneumothorax, either primary or secondary, were enrolled. Patients with bilateral pneumothorax were excluded. Oxygen saturation was measured by pulse oximetry when the patients were in room air. The degree of lung collapse was judged by chest radiography in accordance with the guideline for spontaneous pneumothorax published by The Japan Society for Pneumothorax and Cystic Lung Diseases (http://www.jspcld.jp/en/index.html) as follows: mild, apex of the lung over the clavicle and equivalent state; moderate, middle range between mild and severe; and severe, total collapse and equivalent state. A persistent air leak was defined clinically as an air leak requiring chest drainage for more than 7 days. Duration of chest drainage was defined as the interval between insertion of the chest tube and its removal. After measurement of intrapleural pressure, the pneumothorax was treated conventionally by observation with rest, oxygenation, aspiration, chest tube drainage, chemical pleurodesis, or surgery.

### Needle puncture and measurement of intrapleural pressure

The intrapleural pressure was measured with the patient in the lateral position and the affected side up. The thoracic cavity was punctured using a 16-gauge needle at a site identified as appropriate on chest radiography or CT; this was usually on the lateral side at the 6th or 7th intercostal space. The needle used to puncture the thoracic cavity was connected to the manometer. The puncture needle was held in the operator’s hand for about 30 s while intrapleural pressure was measured.

Industrial equipment designed to measure gas pressure (DHM-01-4kP, 75 × 135 × 35 mm, 212 g, Kobata Gauge Mfg. Co., Ltd., Osaka, Japan, http://www.kobata.co.jp) with high-speed sampling (10 milliseconds) and precision (±0.5% full scale) was modified for use with this system (Additional file 3: Figure S3). A patent is pending for this system. Air pressure can be measured continuously in real time over a range of±4000 Pa when using this system. The measurements are shown as a digital display on the front panel (a liquid crystal screen, 128 × 64 dots). The equipment is linked to a computer with visualization software (HM Viewer, Kobata Gauge Mfg. Co., Ltd.), so the measurements recorded can be seen as a continuous curve showing the changes in air pressure. Dynamic changes in intrapleural pressure are depicted as a periodic curve during monitoring of the patient’s breathing.

### Clinical course after measurement of intrapleural pressure

Although no specific treatment algorithm was used in this study, the pneumothorax was treated after measurement of intrapleural pressure according to whether the purpose of the treatment was respiratory dysfunction, air leak, or recurrence, regardless of whether the pneumothorax was primary or secondary [4]. The initial management of moderate or severe pneumothorax was usually aspiration or chest tube drainage. Patients who were otherwise in good general health were usually managed conservatively by observation, aspiration, and chest drainage in the outpatient clinic. When a patient was treated by chest drainage on an outpatient basis, a 9-Fr tube was used with a flutter valve and a small bottle for fluid drainage.

### Data acquisition and statistical analysis

The intrapleural pressure was measured during stable breathing for about 30 s, representing 10–15 breathing cycles. Data recorded when patients were coughing were excluded. The pressure values at end-expiration and end-inspiration were recorded prospectively. Each patient’s clinical details and the outcome of spontaneous pneumothorax were retrospectively collected for the statistical analysis. Intrapleural pressure was defined as negative if the pressure was lower than atmospheric pressure. The mean values on end-inspiration and end-expiration were calculated. The continuous data were analyzed using the Student’s *t*-test and the categorical data using the chi-squared test. A *p*-value < 0.05 was considered statistically significant. The statistical analysis was performed using JMP version 13.2.1 software (SAS Institute Inc., Cary, NC, USA).

## Results

Eleven patients (8 men, 3 women; mean age 46.6 [20–69] years) were enrolled in the study between December 2016 and July 2017. Four of the patients had a primary spontaneous pneumothorax and 7 had a secondary spontaneous pneumothorax; 4 patients were experiencing pneumothorax for the first time and 7 were experiencing a recurrence. Nine cases showed moderate collapse of the lung on chest radiography and two showed severe collapse (Table 1). Two patients were treated by observation with oxygenation, one by evacuation of air with needle aspiration, and 8 by chest tube drainage (Table 2).

**Table 1:** Patient demographic and clinical patient characteristics

Case	Type of pneumothorax	Side	First episode or recurrence	Onset (days)	Symptomatic	SpO_2_	Degree of collapse
1	Primary spontaneous	Left	First episode	2	+	100	Moderate
2	Secondary spontaneous	Left	Recurrence	30	+	95	Moderate
3	Secondary spontaneous	Left	Recurrence	1	+	95	Moderate
4	Secondary spontaneous	Right	Recurrence	3	+	94	Moderate
5	Secondary spontaneous	Right	Recurrence	30	+	92	Moderate
6	Secondary spontaneous	Right	Recurrence	1	–	96	Moderate
7	Primary spontaneous	Right	Recurrence	2	+	95	Severe
8	Primary spontaneous	Left	First episode	14	+	97	Severe
9	Secondary spontaneous	Right	First episode	10	+	99	Moderate
10	Secondary spontaneous	Left	First episode	6	+	97	Moderate
11	Primary spontaneous	Right	Recurrence		–	97	Moderate

**Table 2:** Intrapleural pressure values, treatment provided, and clinical outcomes

Case	Intrapleural pressure (cmH_2_O)			Treatment and clinical outcome
	End-inspiration	End-expiration	Mean of end-inspiration and end-expiration pressure	
1	−4.18	−1.12	−2.65	Evacuation of 2200 ml of air full expansion
2	−3.16	0.71	−1.23	Drainage for 4 days
3	−3.37	1.43	−0.97	Drainage for 7 days
4	−0.82	2.04	0.61	Drainage for 7 days
5	−19.78	4.79	−7.50	Observation with oxygenation
6	0.10	2.75	1.43	Drainage for 11 days followed by surgery
7	0.00	5.81	2.91	Drainage for 8 days followed by surgery
8	2.65	6.02	4.34	Drainage for 10 days
9	−1.02	0.82	−0.10	Drainage for 1 days
10	−3.26	0.71	−1.28	Drainage for 4 days
11	−12.24	−6.22	−9.23	Observation

**Fig. 1:**
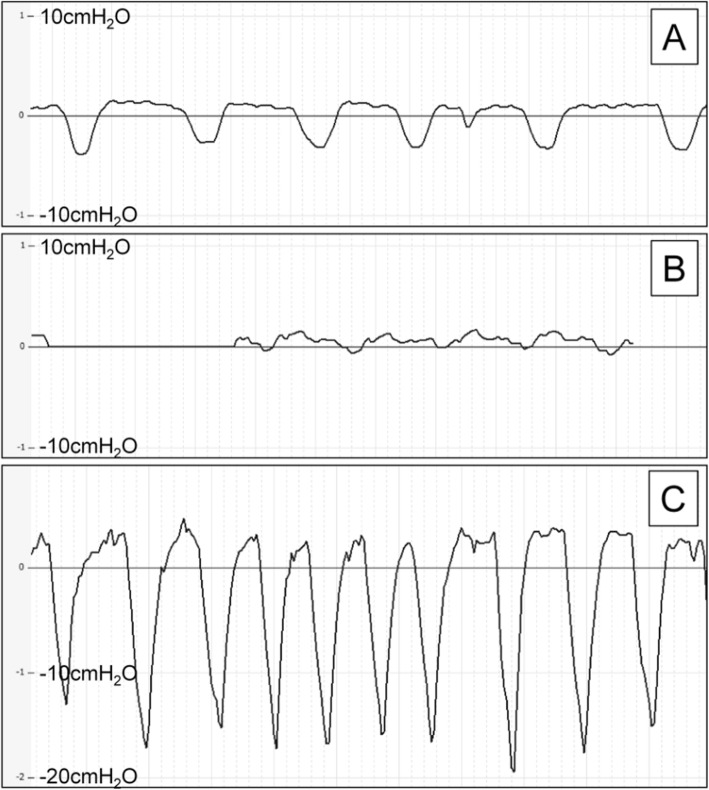
A periodic curve showing intrapleural pressure during breathing in cases 3 (**a**), 4 (**b**), and 5 (**c**)

**Fig. 2:**
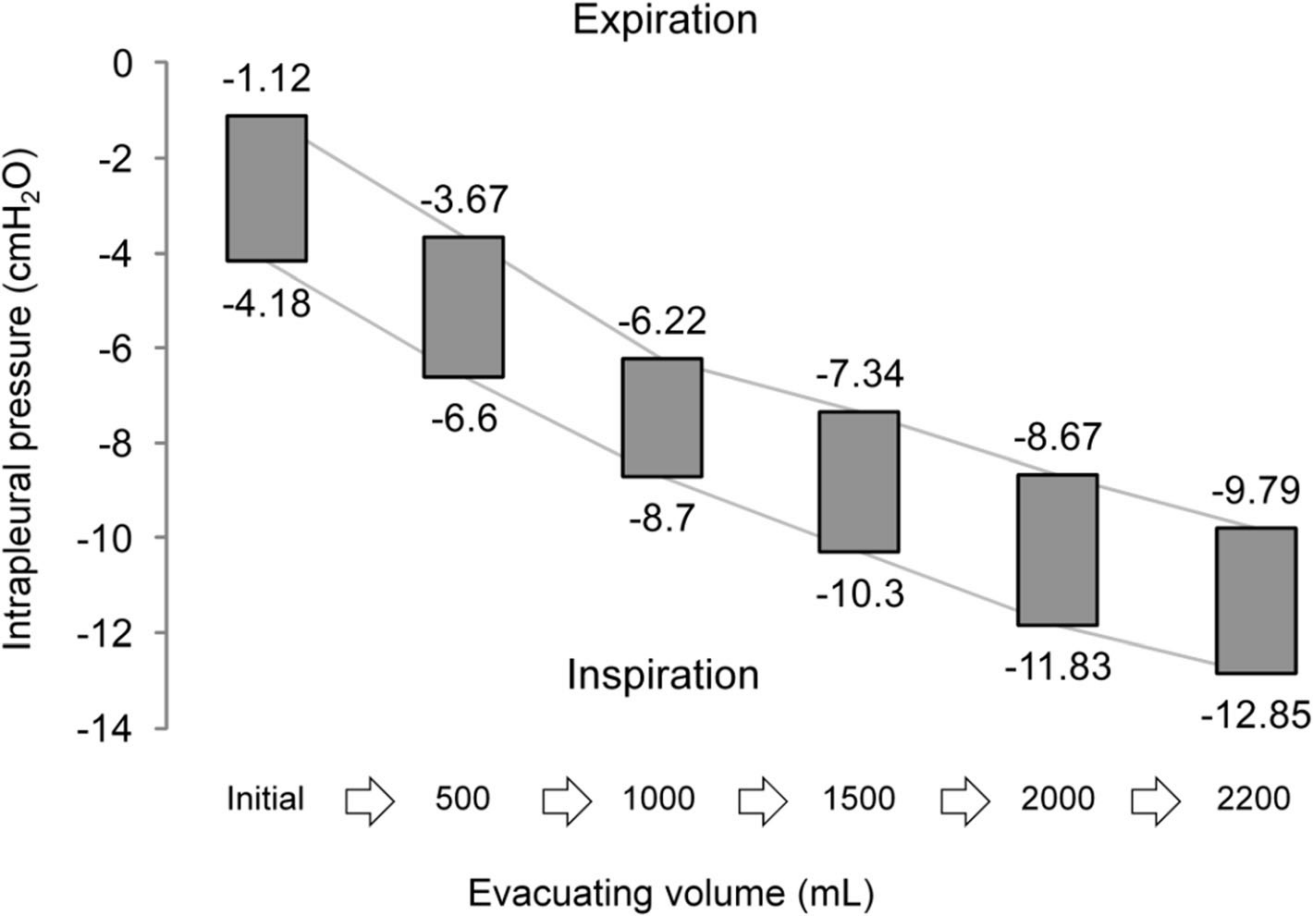
Changes in intrapleural pressure during evacuation of air in case 1

All patients underwent measurements of intrapleural pressure. Figure 1 shows the intrapleural pressure patterns on a periodic curve for 3 patients (cases 3, 4, and 5). These patterns varied widely depending on the state of the pneumothorax. Table 2 shows the intrapleural pressure values recorded at end-expiration and end-inspiration for each patient. The intrapleural pressure was consistently negative during breathing in 2 patients (cases 1 and 11), consistently positive in 3 patients (cases 6, 7, and 8), and negative on end-inspiration and positive on end-expiration in the remaining patients. Figure 2 shows the changes in intrapleural pressure on treatment of aspiration in one of the patients (case 1), in whom the pressure in the thoracic cavity gradually decreased in proportion to the volume of air evacuated.

A comparison of the patients with and without persistent air leak is shown in Table 3. The number of mean intrapleural pressure values on end-inspiration and end-expiration in patients with persistent air leak, defined by a need for more than 7 days of chest drainage, was significantly lower than those in patients without persistent air leak (*p* = 0.020). The number of negative mean pressure recordings in end-inspiration and end-expiration was significantly lower in patients with persistent air leak than in those without persistent air leak (*p* = 0.0060).

**Table 3:** Comparison of intrapleural pressure in patients with and without persistent air leak

Intrapleural pressure	With persistent air leak^a^ (*n* = 5)	Without persistent air leak^a^ (*n* = 6)	*p*-value
Mean of end-inspiration and end-expiration pressure	1.66	−3.67	0.020
Patients with negative mean pressure at end-inspiration and end-expiration, n	1	6	0.0060
Patients with negative pressure at end-inspiration, n	2	6	0.026
Patients with negative pressure at end-expiration, n	0	2	0.15

^a^ Persistent air leak was defined as air leak with chest drainage for more than 7 days

## Discussion

In this study, we demonstrated that intrapleural pressure could be successfully measured and visualized in patients with pneumothorax. Furthermore, we confirmed that the pressure value was a significant predictor of persistent air leak.

The initial management of patients with pneumothorax remains controversial [5]. We previously proposed that treatment should be directed towards treating respiratory dysfunction, stopping the air leak, and avoiding recurrence [4], and we believe that management of pneumothorax involves these three steps. However, to decide on appropriate treatment in the second step, the air leak should be assessed up front, but this is presently impossible [6]. Until now, management decisions have been based on findings on chest radiography and CT and the degree of lung collapse [3]. However, those images are static and cannot determine if air leak is continuing or not [7]. It has been possible to assess intrapleural pressure by observing the movement of water in the sealed chamber of a drainage bag and more recently using a digital system, but only in patients undergoing interventional drainage with a chest tube. According to the consensus statement of the American College of Chest Physicians [8], clinically stable patients with a small pneumothorax should be observed in the emergency department for 3–6 h and then discharged home if a repeat chest radiograph excludes progression of the pneumothorax. Confirmation of collapse of the lung at a single point in time does not provide information on whether an air leak is ongoing. Experienced physicians would agree that more severe symptoms may be correlated with a greater rate of change in intrapleural pressure [9], but may not be directly correlated with the existence of an air leak. To address this issue, we have developed a system for continuous measurement of intrapleural pressure in real time.

Measurement of intrapleural pressure for better understanding of pleural physiology has been described since the 1800s and has been investigated clinically in thoracocentesis for pleural effusion [10–15]. The aim of this technique is to avoid excessive negative pressure in the thoracic cavity that can lead to re-expansion pulmonary edema and secondary pneumothorax [16, 17]. A search of the PubMed database using the terms “pneumothorax”, “intrapleural pressure”, “pleural pressure”, “intrathoracic pressure”, and “manometry” yielded no reports on measurement of intrapleural pressure for pneumothorax. There are few reports on intrapleural pressure and pneumothorax in the English or Japanese literature from the 1970s though to the 1990s. A study that examined pleural pressure in pneumothorax was published by Herrejón A, et al. in 2000 [18]. That study found no relationship between pleural pressure and radiologic size of the pneumothorax and the inspiratory and expiratory pleural pressure values were more negative in patients with spontaneous pneumothorax requiring thoracic drainage for less than 7 days than in their counterparts requiring a longer period of drainage. Their findings are consistent with those of our study.

Measurement of intrapleural pressure has not been adopted in routine clinical practice for patients with pneumothorax or pleural effusion [19], possibly because such measurements are difficult and time-consuming to obtain and not associated with clinical benefit [15]. In this study, we confirmed that our portable and highly accurate visual technique for measurement of intrapleural pressure in patients with pneumothorax in real time is clinically practicable. Importantly, there was a significant relationship between the intrapleural pressure values recorded and persistent air leak.

The main limitations of this study are its retrospective design, the small number of patients included, and the lack of patient homogeneity. However, we believe that our findings warrant prompt introduction of the tool we have developed for assessing air leak in patients with pneumothorax into clinical practice. A prospective study in a larger number of patients is now needed to confirm our present findings and whether or not this measurement system can predict the outcomes of treatment to establish a standard management strategy for patients with pneumothorax.

## Conclusions

The findings of this pilot study show that intrapleural pressure could be successfully measured and visualized in patients with pneumothorax. Whether or not the pressure value is a predictor of persistent air leak needs to be confirmed in the future.

## Supplementary information

**Additional file 1: Figure S1.** (A) A handmade model of the thoracic cavity. (B) A periodic curve showing the change in air pressure using this model. (C) The change in air pressure in a model simulating tension pneumothorax produced by making a small hole in a lung balloon.

**Additional file 2: Figure S2.** Verification using an animal model. (A) The thoracic cavity of a pig was observed by thoracoscopy. (B) A change in air pressure during puncture of the thoracic cavity. The arrow shows the entry of the tip of the needle into the thoracic cavity. (C) A periodic curve showing intrapleural pressure accompanied by pulmonary ventilation.

**Additional file 3: Figure S3.** A needle to puncture thoracic cavity connected to the manometer to measure intrapleural pressure.

## Data Availability

The datasets and related materials within the study can be available from the corresponding author on a reasonable request.

## Abbreviation

CT: Computed tomography
