# Supplementary figures and images for: Measurement of intrapleural pressure in patients with spontaneous pneumothorax: a pilot study
Source: BMC Pulm Med. 2019 Dec 30;19:267. doi: 10.1186/s12890-019-1038-9 (PMC6938002; doi:10.1186/s12890-019-1038-9)

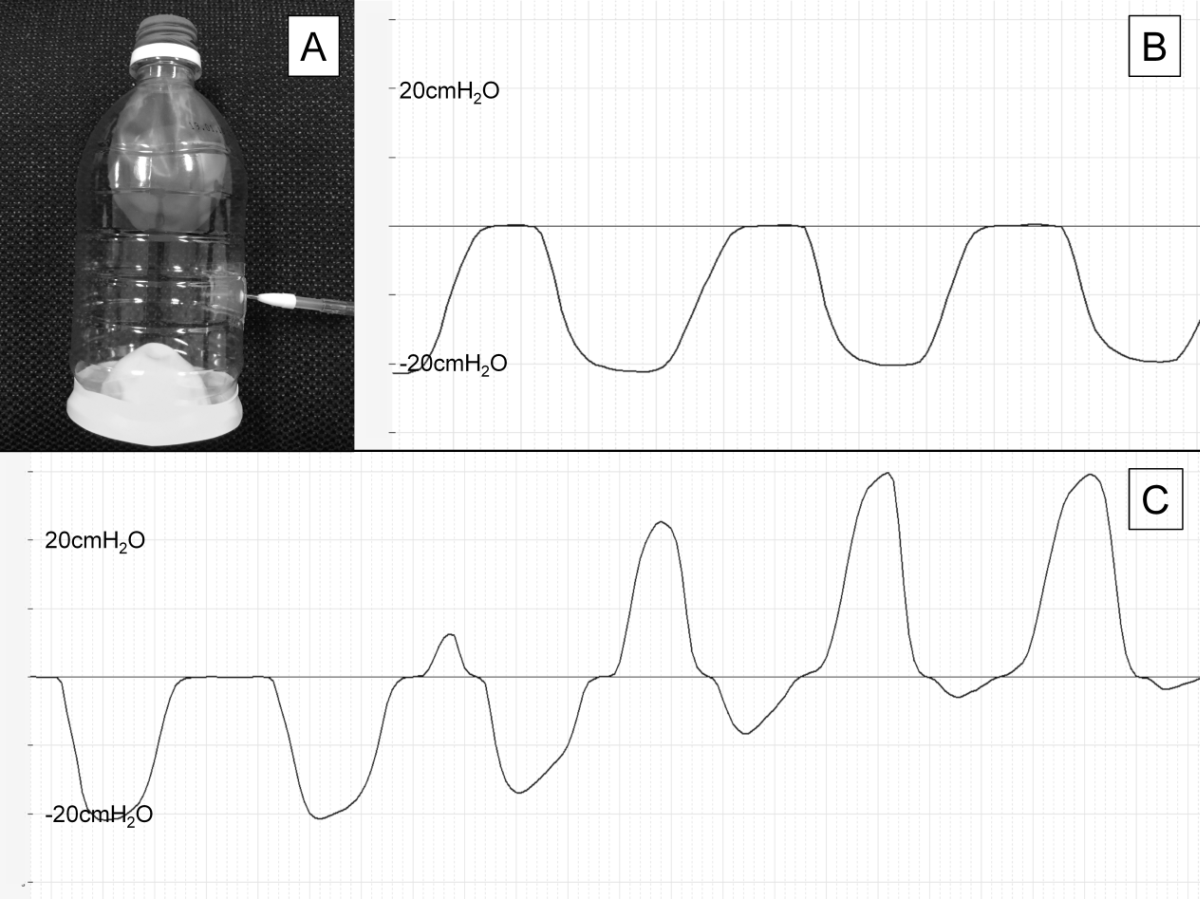

Supplement: Supplementary file 1 — Additional file 1: Figure S1. (A) A handmade model of the thoracic cavity. (B) A periodic curve showing the change in air pressure using this model. (C) The change in air pressure in a model simulating tension pneumothorax produced by making a small hole in a lung balloon. [file 12890_2019_1038_MOESM1_ESM.tif]

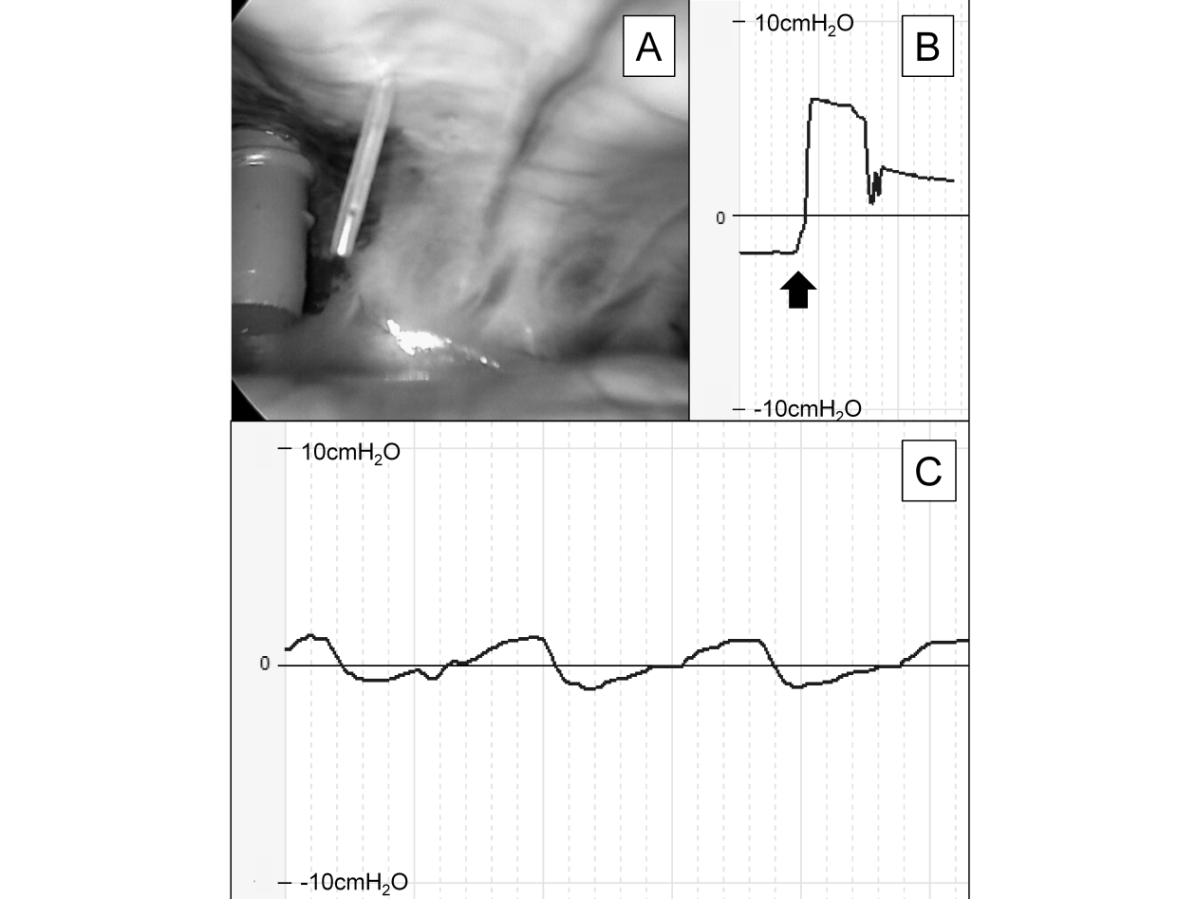

Supplement: Supplementary file 2 — Additional file 2: Figure S2. Verification using an animal model. (A) The thoracic cavity of a pig was observed by thoracoscopy. (B) A change in air pressure during puncture of the thoracic cavity. The arrow shows the entry of the tip of the needle into the thoracic cavity. (C) A periodic curve showing intrapleural pressure accompanied by pulmonary ventilation. [file 12890_2019_1038_MOESM2_ESM.tif]

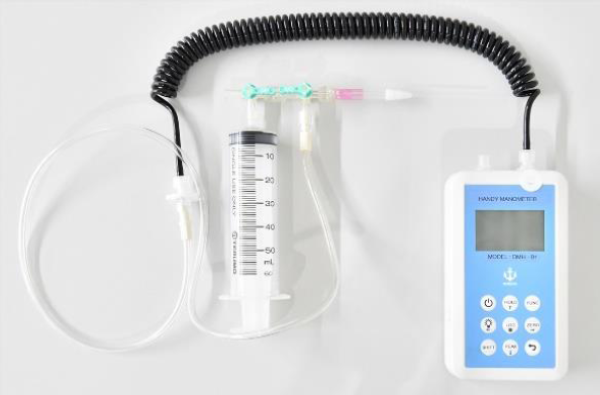

Supplement: Supplementary file 3 — Additional file 3: Figure S3. A needle to puncture thoracic cavity connected to the manometer to measure intrapleural pressure. [file 12890_2019_1038_MOESM3_ESM.tif]
